# Supplementary material for: Dynamic gene regulation by nuclear colony-stimulating factor 1 receptor in human monocytes and macrophages
Source: Nat Commun. 2019 Apr 26;10:1935. doi: 10.1038/s41467-019-09970-9 (PMC6486619; doi:10.1038/s41467-019-09970-9)
Supplement: Supplementary file 1 — Supplementary Information [file 41467_2019_9970_MOESM1_ESM.pdf]

Supplementary informations

Dynamic gene regulation by nuclear colony-stimulating factor 1 receptor in human monocytes and macrophages

Bencheikh L et al.

Laura Bencheikh *et al*, Supplementary Figure 1

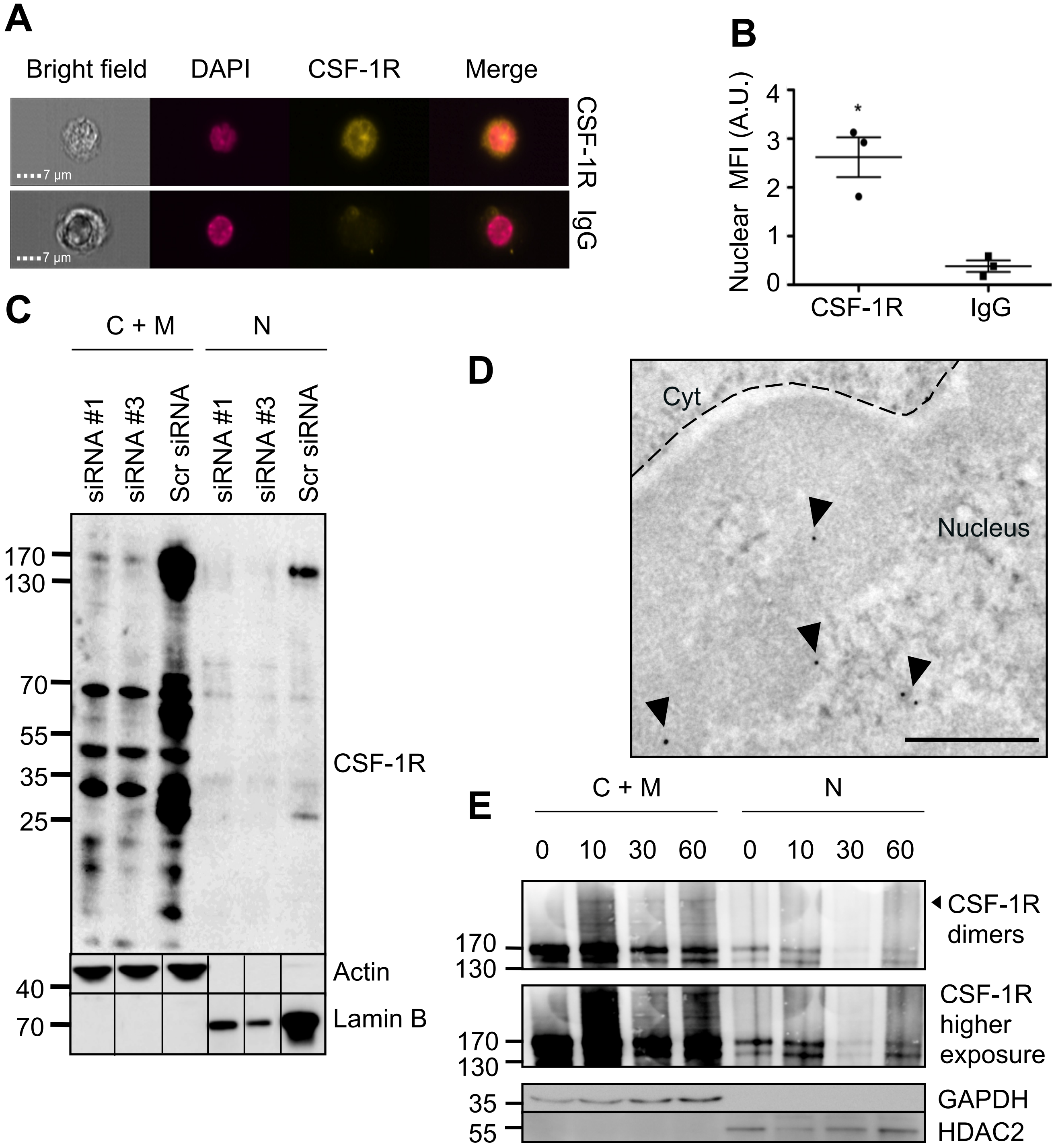

Supplementary Figure 1: A fraction of CSF-1R is located in the nucleus of human monocytes.

A,B. Monocytes were fixed and stained for CSF-1R (anti-Cter sc-692) or a non-relevant antibody (IgG) and Dapi, followed by imaging flow cytometry (Amnis); **A.** representative image (DAPI in purple and CSF-1R in yellow, scale : 7μm); **B.** quantification of nuclear CSF-1R (mean +/- SEM of 3 independent experiments, MFI : mean fluorescence intensity, A.U. : arbitrary units, *P<0.05). **C.** Monocytes were transfected with CSF-1R specific (#1 and 3) or control (Co) siRNAs; 24 hours later, cytoplasmic plus membrane (C+M) and nuclear (N) proteins were extracted and separated for immunoblotting with CSF-1R, Lamin B (nucleus marker) and actin (cytoplasmic marker) antibodies (n = 1). **D.** Electron microscopy analysis of monocytes stained with an anti-CSF-1R Nter (sc-365719) antibody (scale: 500nm, n = 1). **E.** Monocytes were fractionated into cytoplasmic plus membrane (C+M) and nucleus (N) before and after CSF-1 stimulation (100ng/mL for indicated times in minutes), and proteins extracted from these fractions in the absence of DTT and boiling were analyzed by immunoblotting for CSF1-R, histone deacetylase 2 (HDAC2, nuclear protein) and GAPDH (cytplasmic protein) (n = 2). Two exposures of CSF-1R blot are shown.

Laura Bencheikh *et al*, Supplementary Figure 2

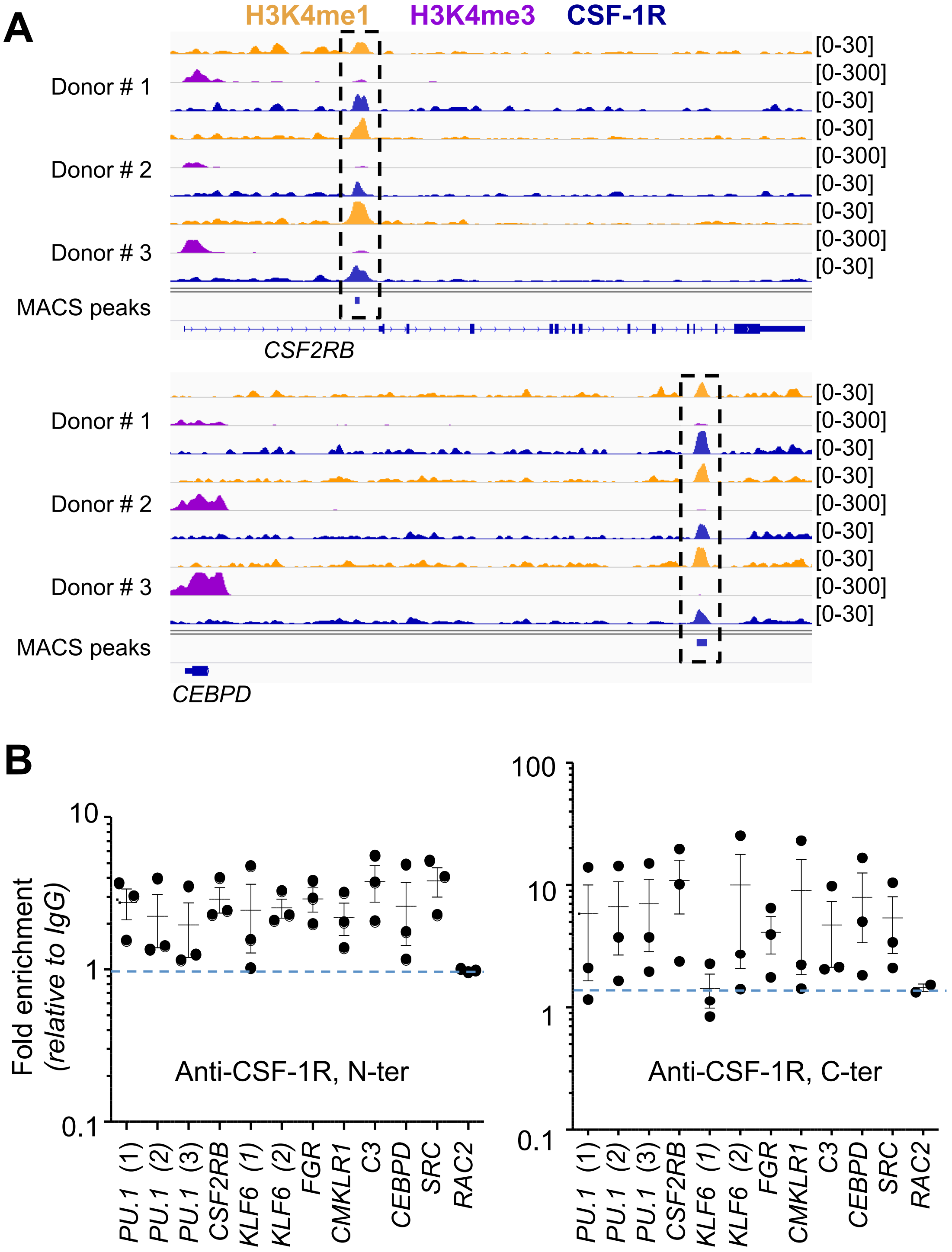

Supplementary Figure 2: **CSF-1R is recruited to the chromatin in human monocyte nucleus**.

A. Peak calling for CSF-1R (blue), H3K4me1 (orange) and H3K4me3 (purple) ChIP-seq on *CSFR2B* and *CEBPD* genes in the three healthy donor monocytes (TSS: transcription starting site, TTS: transcription termination site). **B.** Chromatin was immunoprecipitated from monocytes with the anti-N-ter (sc-46662) (left panel) or the anti-C-ter (sc-692) (right panel) CSF-1R antibody or control IgG followed by qPCR directed against *PU.1 [promoter (-1739), intron 4.1 (20202), intron 4.2 (21399)]*, *CSF2RB (7581)*, *KLF6 (1) (-17610)*, *KLF6 (2) (-23783)*, *FGR (1599), CMKLR1 (33519)*, *C3(3024)*, *CEBPD (-25567)* and *SRC (72944)* genes, using *RAC2* as a control whose level if figured as a hatched blue bar (normalization to IgG, mean +/- SEM of 3 independent experiments). Results are normalized to IgG (mean +/- SEM of 3 independent experiments).

Laura Bencheikh *et al*, Supplementary Figure 3

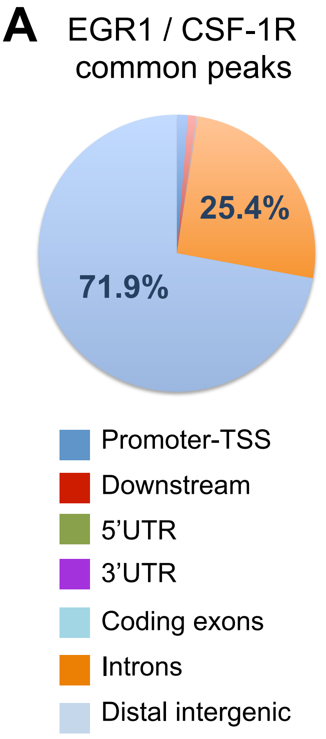

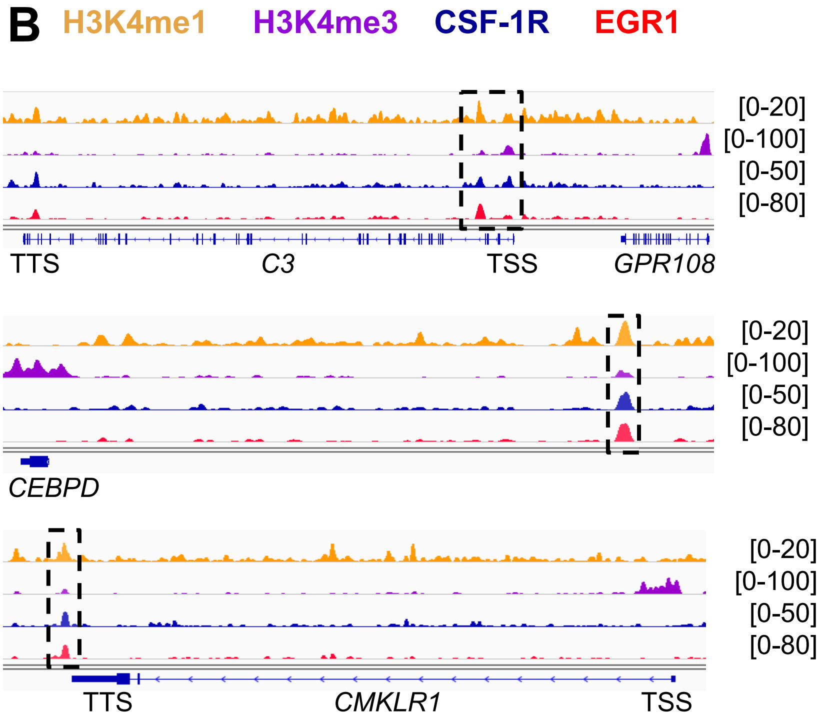

C

Motif	Transcription factor	Target sequence with motif	*P* value
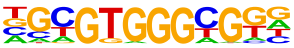	EGR2	22.9%	$1e^{-194}$
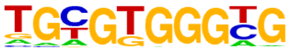	EGR1	42.9%	$1e^{-167}$
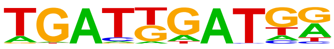	HOXA1	24.8%	$1e^{-85}$
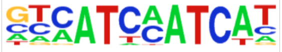	HOXA2	14.9%	$1e^{-85}$

Supplementary Figure 3: **CSF-1R interacts with EGR1 to down-regulate gene expression**.

A. Repartition of CSF-1R peaks detected on EGR1 motif and common to three healthy donors, to be compared to genome organization shown on Figure 3b. **B.** Peak calling of ChIP-Seq experiments performed with H3K4me1 (orange), H3K4me3 (purple), EGR1 (orange), and CSF-1R (dark blue) antibodies on *C3, CEBPD* and *CMKLR1* genes in human monocytes of healthy donors (TSS, transcription starting site; TTS, transcription termination site). **C.** Motif analysis of EGR1 peaks common to two healthy donors.

Laura Bencheikh *et al*, Supplementary Figure 4

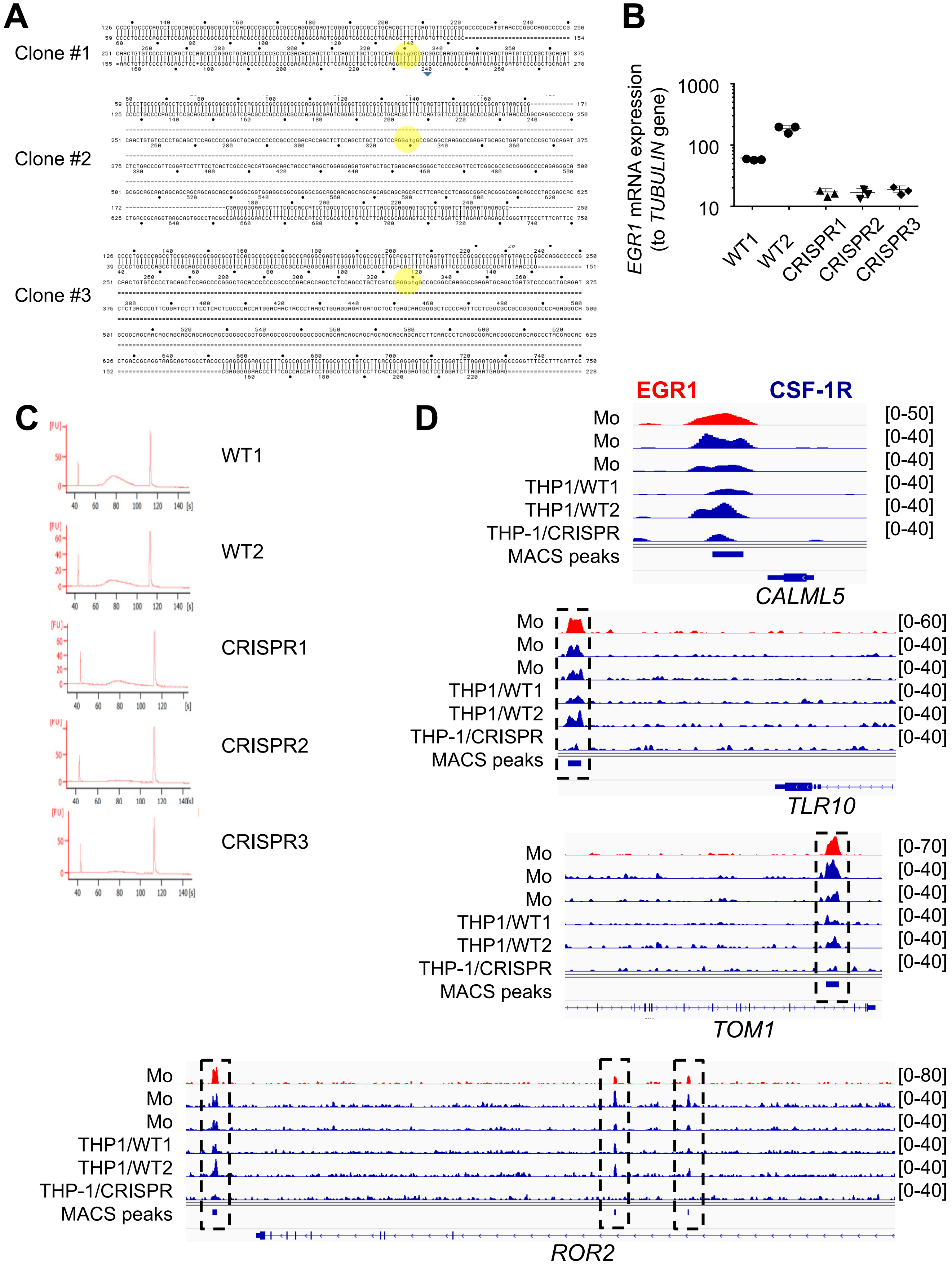

Supplementary Figure 4: EGR1 is involved in CSF-1R recruitment on chromatin in THP-1 cells.

A. Sequencing of *EGR1* exon 1 in 3 THP-1 clones showing CRISPR-Cas9 mediated deletion. Sequences aligned against wild-type *EGR1* sequence. **B**. mRNA expression (RT-qPCR) of *EGR1* gene in wild-type and *EGR1*-deleted clones (mean +/- SD of 3). **C**. Migration profile (Agilent Bioanalyzer) of Illumina libraries built using DNA immunoprecipitated with anti-CSF-1R antibody (sc-46662) in wild-type (WT) and EGR1-deleted clones (CRISPR). **D**. Peak calling on *CALML5*, *TLR10*, *TOM1* and *ROR2* genes for EGR1 in one monocyte sample and CSF-1R in two monocyte samples (Mo), two wild-type (WT) THP-1 clones and the pool of 3 EGR1-deleted THP-1 clones (CRISPR).

Laura Bencheikh *et al*, Supplementary Figure 5

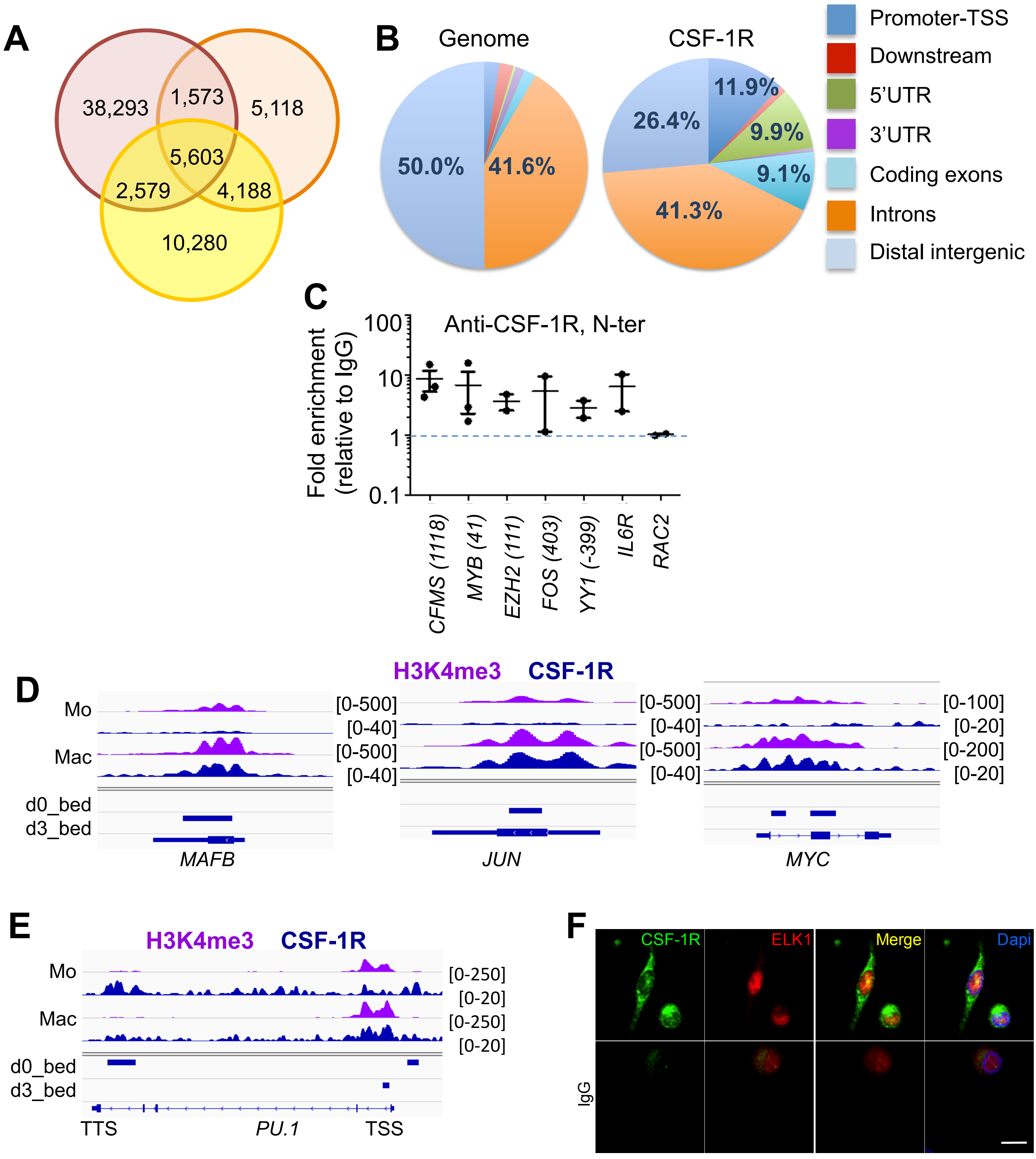

Supplementary Figure 5: Differential CSF-1R chromatin localization in CSF-1-induced macrophages.

A. Venn diagram of peak calling (input normalization) from ChIP-seq experiment (anti-CSF-1R sc-46662) performed on monocytes differentiated 3 days with 100ng/mL CSF-1 from 3 donors. **B**. Repartition of the ChIP-seq peaks common to the 3 donors on the genome compared to genome of reference (Genome). **C**. Monocytes were stimulated 3 days with 100ng/mL CSF-1. Chromatin was immunoprecipitated with CSF-1R antibody (anti-N-ter sc-46662) or control IgG followed by qPCR directed against *CFMS, MYB, EZH2, FOS*, YY1 and IL6R genes (normalization to IgG, mean +/- SEM of 2 independent experiments). RAC2 is a negative control whose level if figured as a hatched blue bar. **D**. Peak calling for H3K4me3 (purple) and CSF-1R (blue) on *MAFB, JUN, MYC* **E**. and *PU.1* genes in monocytes (Mo) compared to CSF-1 (3 days) differentiated-macrophages (Mac) (TSS : transcription starting site; TTS : transcription termination site). **F**. Monocytes were treated 3 days with 100ng/mL CSF-1, fixed and stained for CSF-1R (green), ELK1 (red) or control IgG and Dapi (blue) followed by confocal imaging (n = 2, scale : 10µm).

Laura Bencheikh *et al*, Supplementary Figure 6

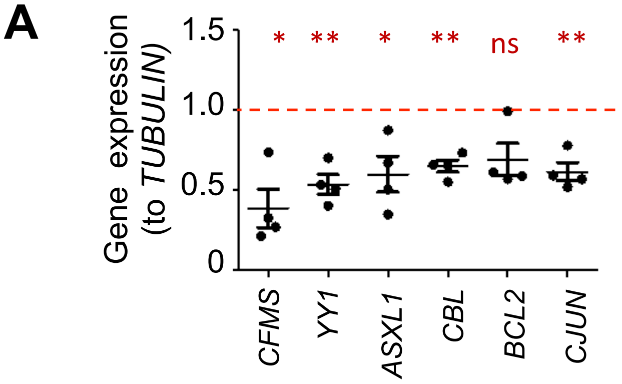

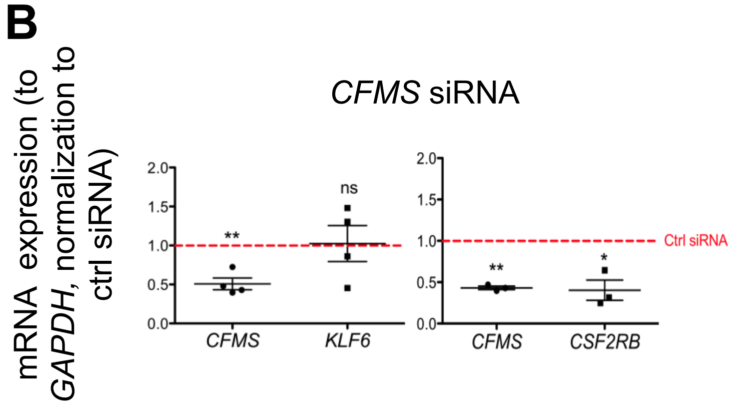

Supplementary Figure 6: Differential gene regulation by CSF-1R in CSF-1-induced macrophages.

Monocytes were treated with 100 ng/ml CSF-1 for 2 days and transfected during 48h with scrambled (SCR) or *CFMS*-specific siRNA **A.** RT-qPCR analysis of *CFMS, YY1, ASXL1, CBL, BCL2* and *CJUN* genes **B.** RT-qPCR analysis of *CFMS*, *KLF6* and *CSF2RB* gene expression. Results in the two panels are expressed as a mean +/- SEM of 3 to 4 independent experiments, normalization to control siRNA indicated as a red hatched line, *p<0.05, **P<0.01, ns : non significant.

Laura Bencheikh *et al*, Supplementary Figure 7

Uncropped blots related to Figure 1e

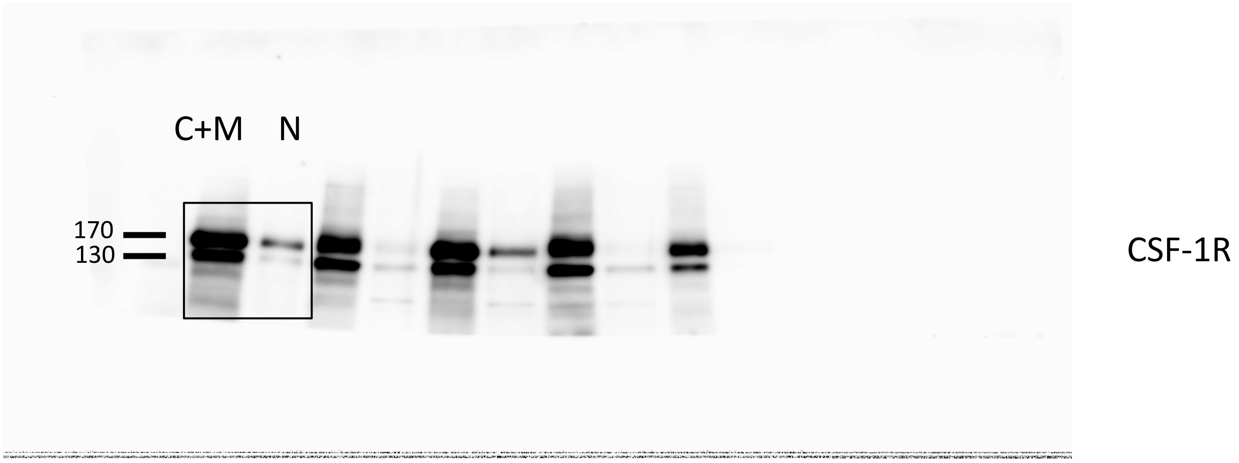

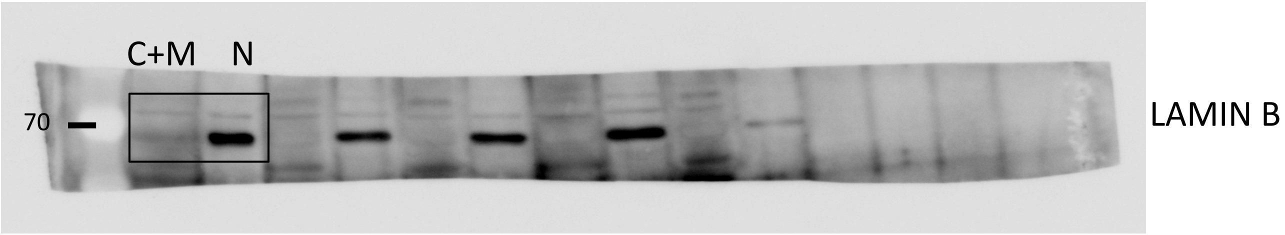

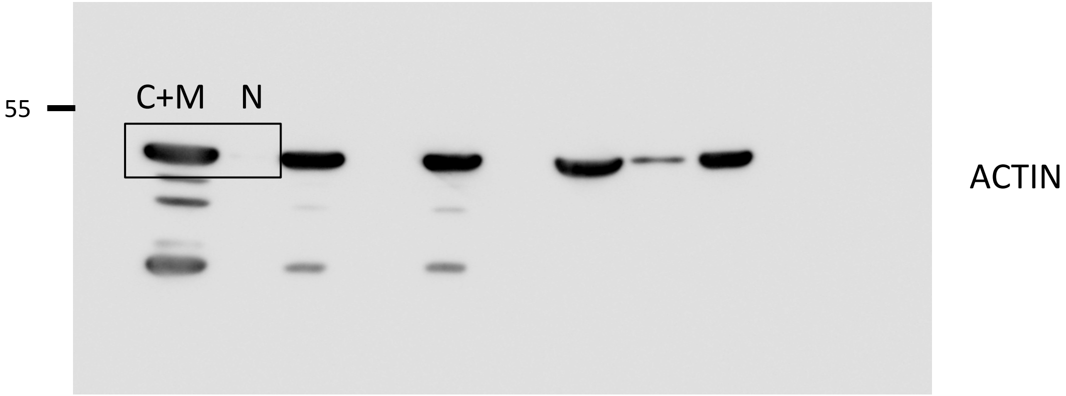

Laura Bencheikh *et al*, Supplementary Figure 7

Uncropped blots related to Figure 4e

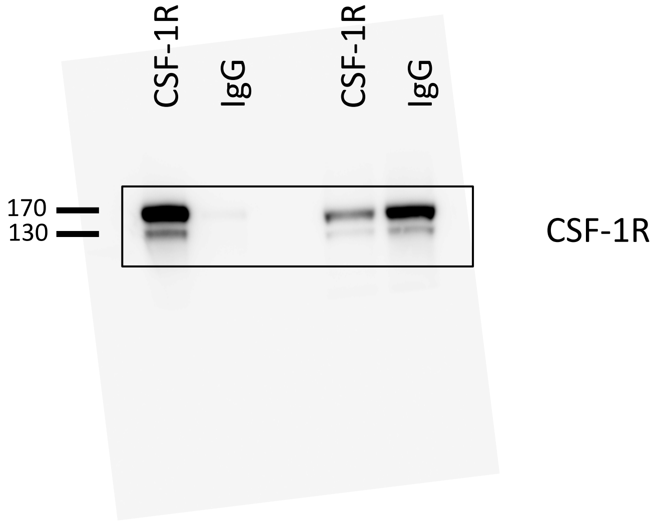

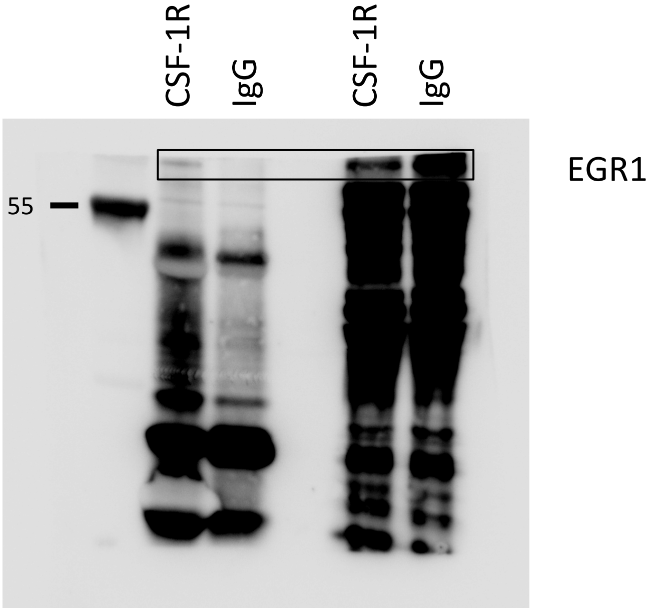

Laura Bencheikh *et al*, Supplementary Figure 7

Uncropped blots related to Figure 6a

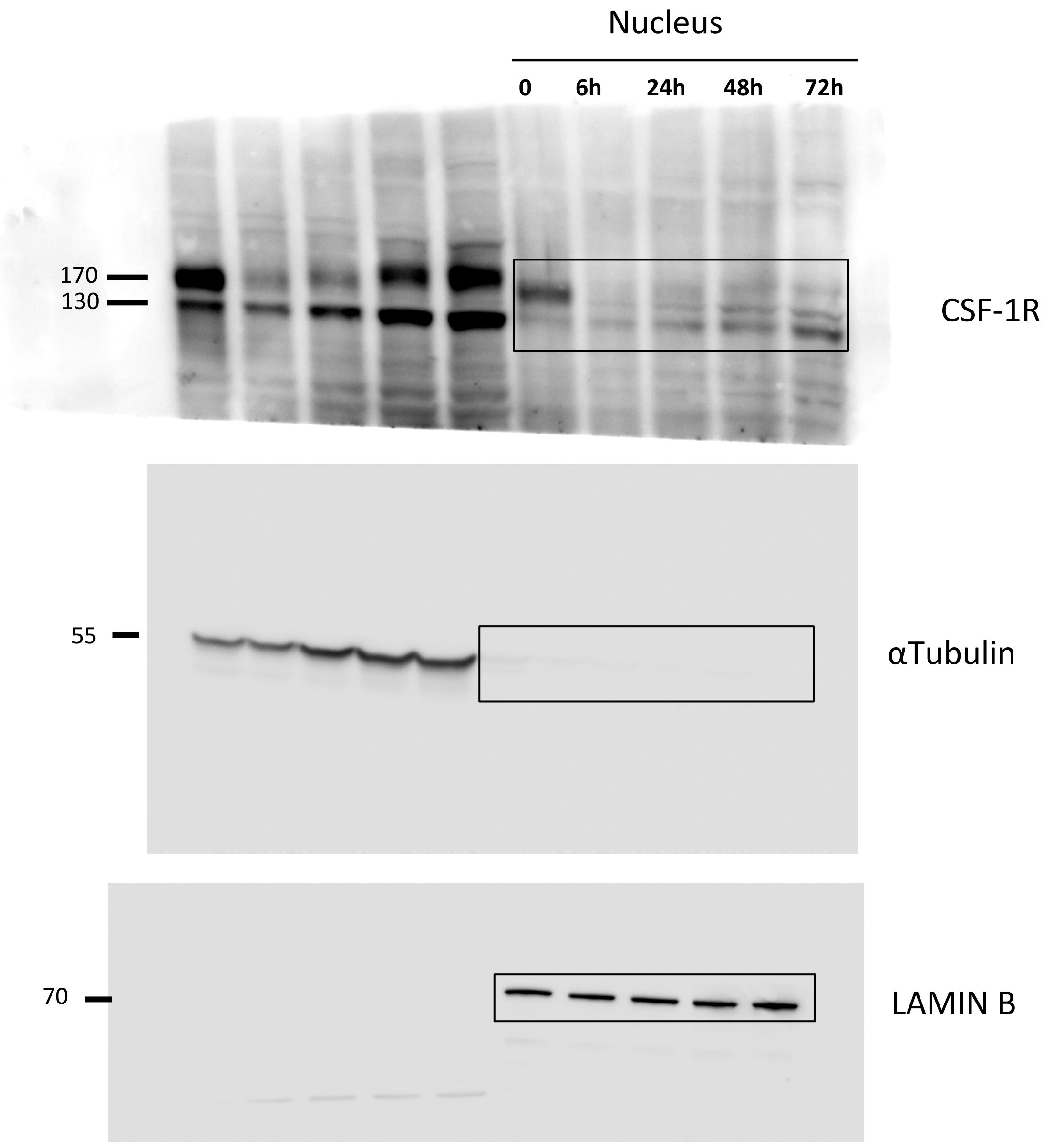

Laura Bencheikh *et al*, Supplementary Figure 7

Uncropped blots related to Figure 7d

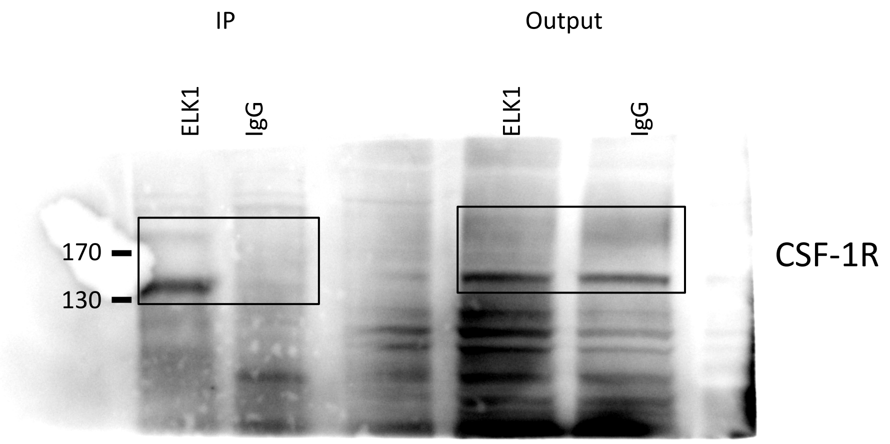

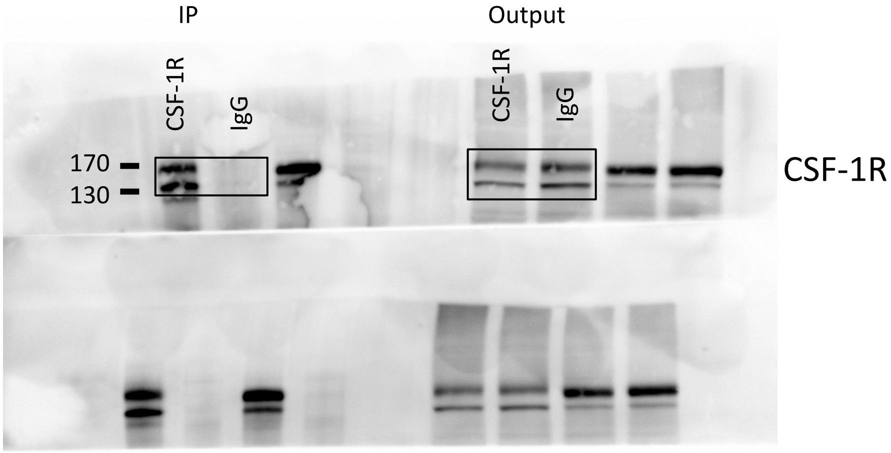

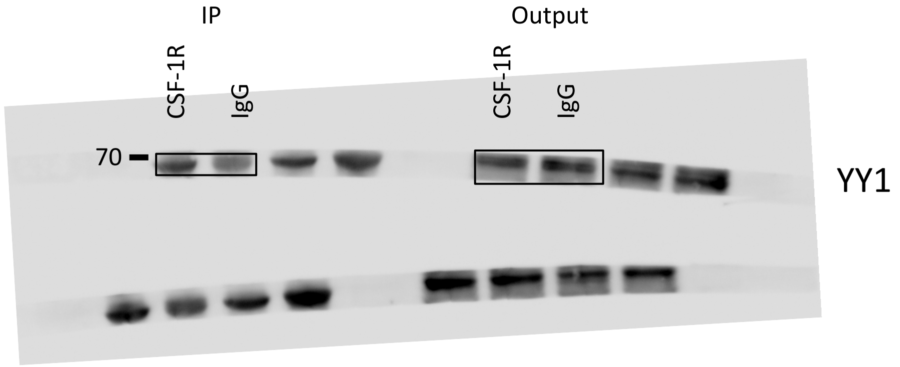

Laura Bencheikh *et al*, Supplementary Figure 7

Uncropped blots related to Figure 9a

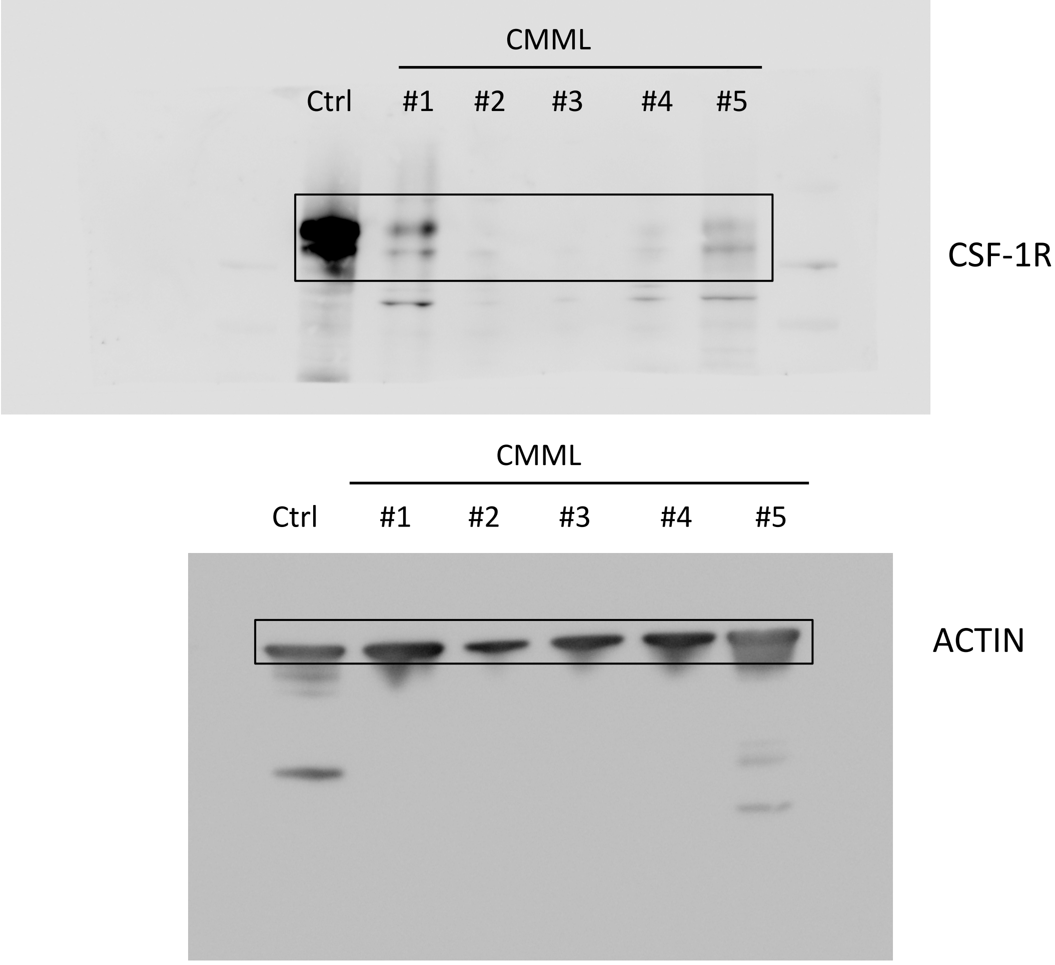

Supplementary Figure 7: Uncropped scans of key immunoblots.

Supplementary Tables

Supplementary Table 1: Enrichment of Gene Ontology biological process terms in ChIP hits with CSF-1R in monocytes

Go term ID	GO biological process	Fold Enrichment	P-value
GO:0036179	osteoclast maturation	10.38	3.48 e-7
GO:0010934	macrophage cytokine production	10.33	3.25 e-5
GO:0050902	leukocyte adhesive activation	10.29	3.32 e-3
GO:2000669	negative regulation of dendritic cell apoptotic process	9.96	2.11 e-6
GO:0036035	osteoclast development	9.13	6.41 e-8
GO:0071674	mononuclear cell migration	8.37	3.86 e-4
GO:0033159	negative regulation of protein import into nucleus, translocation	7.12	2.33 e-5
GO:2000510	positive regulation of dendritic cell chemotaxis	6.19	5.06 e-4
GO:0033235	positive regulation of protein sumoylation	5.27	1.28 e-10
GO:0061082	myeloid leukocyte cytokine production	3.74	1.66 e-3

Supplementary Table 2: Enrichment of Gene Ontology biological process terms in ChIP hits with CSF-1R in monocytes exposed to CSF-1 for 3 days

Go term ID	GO biological process	Fold Enrichment	P-value
GO:0010847	regulation of chromatin assembly	12.92	1.76 e-3
GO:0000389	mRNA 3'-splice site recognition	8.67	1.31 e-3
GO:0072369	regulation of lipid transport by positive regulation of transcription from RNA polymerase II promoter	8.41	3.79 e-4
GO:0072363	regulation of glycolysis by positive regulation of transcription from RNA polymerase II promoter	7.29	2.44 e-3
GO:0046833	positive regulation of RNA export from nucleus	6.86	1.01 e-2
GO:0072367	regulation of lipid transport by regulation of transcription from RNA polymerase II promoter	5.66	2.30 e-4
GO:0036179	osteoclast maturation	5.13	3.29 e-3
GO:0036035	osteoclast development	3.69	1.26 e-2
GO:0090314	positive regulation of protein targeting to membrane	3.18	9.26 e-4
GO:0008334	histone mRNA metabolic process	2.47	5.68 e-4

Supplementary Table 3: Enrichment of Gene Ontology biological process terms in ChIP hits with CSF-1R in monocytes exposed to CSF-1 for 6 hours

Go term ID	GO biological process	Fold Enrichment	P-value
GO:0071677	positive regulation of mononuclear cell migration	110.34	2.52E-06
GO:0035691	macrophage migration inhibitory factor signaling pathway	26.63	8.27E-07
GO:0038145	macrophage colony-stimulating factor signaling pathway	11.18	9.12E-04
GO:0050765	negative regulation of phagocytosis	7.41	4.13E-05
GO:2000785	regulation of autophagic vacuole assembly	7.71	2.94E-04
GO:0006890	retrograde vesicle-mediated transport, Golgi to ER	4.55	1.92E-04
GO:0033235	positive regulation of protein sumoylation	3.66	6.74E-04
GO:0002246	wound healing involved in inflammatory response	3.66	2.95E-02
GO:0000184	nuclear-transcribed mRNA catabolic process, nonsense-mediated decay	2.86	8.78E-05
GO:0019083	viral transcription	2.83	5.87E-06

Supplementary Table 4: Primer sequences used for RT-qPCR analyses

Gene	Forward	Reverse
CFMS	5'-GCCCCCCATCACCTCACT-3'	5'-GTGTTTTGGAAGGTAGCGTTGTT-3'
LUCIFERASE	5'-CAGCTGCACAAAGCCATGA-3'	5'-CGATATGTGCGTCGGTAAAGG-3'
B2M	5'-ACACAACTGTGTTCACTAGC-3'	5'-CAACTTCATCCACGTTCACC-3'
GAPDH	5'-AAGGTCGGAGTCAACGGGT-3'	5'-AGAGTTAAAAGCAGCCCTGGTG-3'
TUBULIN A	5'-TCCAGATTGGCAATGCCTG-3'	5'-GGCCATCGGGCTGGAT-3'
RENILLA	5'-AATTTGCAGCATATCTTGAACCATT-3'	5'-GGATTTCACGAGGCCATGAT-3'
HPRT	5'-GGACAGGACTGAACGTCTTGC-3'	5'-CTTGAGCACACAGAGGGCTACA-3'
RPL32	5'-TGTCCTGAATGTGGTCACCTGA-3'	5'-CTGCAGTCTCCTTGCACACCT-3'
PU.1	5'-GGAGAGCCATAGCGACCATTACT-3'	5'-CGGCGAAGCTCTCGAACTC-3'
BCL2	5'-AACTGTACGGCCCCAGCAT-3'	5'-GCCAAACTGAGCAGAGTCTTCAG-3'
EZH2	5'-CGCTGACCATTGGGACAGTA-3'	5'-TTGGAGCCCCGCTGAATA-3'
ASXL1	5'-CCGCGCGCCTGGTA-3'	5'-CTGCAGAATCTGTTTTGGTGTCAT-3'
CBL	5'-GAAGTGCTGGAAGCTCATGGA-3'	5'-CGCCAGCTTTGGGTTCTG-3'
CSF2RB	5'-TACAACGACTACACCAGCCACAT-3'	5'-AGCCGCTGGGCATCCT-3'
CEBPD	5'-GGAGATGCAGCAGAAGTTGGT-3'	5'-CGCGCTGGTGCAGCTT-3'
SRC	5'-GGACAGCCCCTGCCTTCTA-3'	5'-GGCATCCTTGGGCTTGCT-3'
KLF6	5'-TGCACGAGACCGGCTACTTC-3'	5'-TAGGCAGGTCTGTTGCCAGTAC-3'
YY1	5'-GGCTGCGTCTCAGGTTTCC-3'	5'-CCACCGTTGGGAGGATGTC-3'
C3	5'-GGGAGTCCCATGTACTCTATCATCA-3'	5'-TGGGCCTCCAGCACCAT-3'
FGR	5'-CCATCAACCCTGGCTTCCT-3'	5'-CACCCCAATCCCTGACACA-3'
CJUN	5'-AACGACCTTCTATGACGATGCC-3'	5'-TGTAGCCATAAGGTCCGCTCTC-3'
EGR1	5'-TGATGTCCCCGCTGCAG-3'	5'-GTCCATGGTGGGCGAGTG-3'

Supplementary Table 5: Primer sequences used for ChIP-qPCR analyses

Gene	Forward	Reverse
PU.1 promoter	5'-CTCCACCCCCCAGAAAAGAT-3'	5'-GCGCTGGGAAAACAAAAAAG-3'
PU.1 intron 4-1	5'-GGCTGAGACAGGAGAATTGCTT-3'	5'-GGCACGATCTCGGCTCACT-3'
PU.1 intron 4-2	5'-GGAGGTGGAGGTTGCAGTGA-3'	5'-GTTTTTGAGATGGAGTTGCTCTTGT-3'
CSF2RB intron1	5'-GTTTGCATGGGCATCTTGAGT-3'	5'-CAAGTGTGGCTCCTTTTCTTCTG-3'
KLF6-18kb	5'-TGTGTGCCTCTGTGTGTATGTG-3'	5'-CTTACCCACACAGAGACACACAGAC-3'
KLF6-24kb	5'-TCACAGACACACATGCGTACAC-3'	5'-GTCTCTCTGTGTGTCTGCTGCATA-3'
C3 intron 4	5'-TCCGTGGATTCTGCTTTCAGT-3'	5'-CGTCATGATCCCGATATTCAGAT-3'
FGR intron 2	5'-AGGCAAACTCACCCATACTTAGAG-3'	5'-GTCCTATTGCTTTGATATGTTGTGTTTC-3'
CMKLR1	5'-CTACCACAGAATTAGCAAAAGAGTAGCT-3'	: 5'-CGCCTAGCCATCCATCCA-3'
CEBPD	5'-CCCGAGAGGATTCAGACTGG-3'	5'-ACACACTAGGAGGTTCATTGC-3'
SRC	5'-GACTCTGCTCATGCCACGTATTT-3'	5'-GCCAGAAGAAGTGGGAGAAGAC-3'
CFMS intron 1	5'-CCCCAACACCTGCAATTGA-3'	5'-CCTGAGCAAGCCTCTGATACTCTT-3'
MYB TSS	5'-GCCGCTCCAGAGACTGATG-3'	5'-CCCAACGTCCGGATACATTT-3'
EZH2 TSS	5'-GGAGACACAGTCCGTCCTTTG-3'	5'-ACGGGACAGACACAAGTTGGT-3'
FOS intron 1	5'-GGGATAGCCTCTCTTACTACCACTCA-3'	5'-ACAGGCGAGCCCATGCT-3'
YY1 TSS	5'- GGCTGCGTCTCAGGTTTCC -3'	5'- CCACCGTTGGGAGGATGTC -3'
ILR6 intron1	5'- CATGGTAGCATGTACCTGTAGTCTCA -3'	5'- TGGTTCACTGCAACTTCTAATTCC -3'
RAC2	5'- ATCTCAGGGCATCCCAGTTG -3'	5'- GGTGGGAGATGGGTAGGTACCT -3'

Supplementary Table 6

**CMML patient characteristics**	N = 28
Mean age in years (range)	80.3 (60-96)
Sex ratio M/F	15/13
CMML 0/CMML 1/CMML 2 (WHO criteria)	10/14/3
Proliferative/dysplastic (WHO criteria)	14/14
Leucocyte mean.10^9/L (range)	27.3 (2.1-167.4)
Monocyte mean.10^9/L (range)	8.1 (1.0-110.0)
Platelet count mean.10^9/L (range)	146 (23-597)
Hemoglobin level mean g/dL (range)	11.5 (7.7-15.9)
Karyotype (normal/abnormal/not done)	24/3/1
**Mutations (mutated/done)**	
TET2	20/27 (74%)
SRSF2	11/24 (46%)
ASXL1	10/24 (42%)
RUNX1	3/24 (12.5%)
NRAS	2/22 (9%)
KRAS	2/25 (8%)
CBL	2/25 (8%)
