# Supplementary material for: Dynamic gene regulation by nuclear colony-stimulating factor 1 receptor in human monocytes and macrophages
Source: Nat Commun. 2019 Apr 26;10:1935. doi: 10.1038/s41467-019-09970-9 (PMC6486619; doi:10.1038/s41467-019-09970-9)
Supplement: Supplementary file 3 — Description of Additional Supplementary Files [file 41467_2019_9970_MOESM3_ESM.pdf]

Description of Additional Supplementary Files

File Name: Supplementary Data 1

Description: List of 4,980 common CSF-1R broad peaks identified using MACS2 algorithm in monocytes sorted from three healthy donors.

File Name: Supplementary Data 2

Description: Intersection with Irreproducible Discovery Rate (IDR) analysis identified a set of 2,303 common peaks.

File Name: Supplementary Data 3

Description: List of 3,542 EGR1 common peaks identified using MACS2 algorithm in monocytes sorted from two healthy donors.

File Name: Supplementary Data 4

Description: List of 2,014 EGR1 and CSF-1R common peaks identified using MACS2 algorithm in monocytes sorted from healthy donors.

File Name: Supplementary Data 5

Description: List of 1,575 EGR1, CSF-1R and H3K4me1 common peaks identified using MACS2 algorithm in monocytes sorted from healthy donors.

File Name: Supplementary Data 6

Description: List of 5,603 common CSF-1R broad peaks identified using MACS2 algorithm in CSF-1-differentiated macrophages from three healthy donors.

File Name: Supplementary Data 7

Description: List of 1,192 common CSF-1R broad peaks identified using MACS2 algorithm in monocytes and macrophages.

File Name: Supplementary Data 8

Description: List of 2,975 common CSF-1R and H3K4me1 peaks identified using MACS2 algorithm in monocytes.

File Name: Supplementary Data 9

Description: List of 1,605 common CSF-1R and H3K4me3 peaks identified using MACS2 algorithm in monocytes.

File Name: Supplementary Data 10

Description: List of 1,925 common CSF-1R and H3K4me1 peaks identified using MACS2 algorithm in macrophages.

File Name: Supplementary Data 11

Description: List of 4,896 common CSF-1R and H3K4me3 peaks identified using MACS2 algorithm in macrophages.

File Name: Supplementary Data 12

Description: List of 2,388 CSF-1R peaks identified using MACS2 algorithm in monocytes after 6 hours of CSF-1 exposure.

File Name: Supplementary Data 13

Description: List of 2,157 common CSF-1R and H3K4me3 peaks identified using MACS2 algorithm in monocytes after 6 hours of CSF-1 exposure.

File Name: Supplementary Data 14

Description: List of 680 common CSF-1R peaks identified using MACS2 algorithm in monocytes at day0 and after 6 hours of CSF-1 exposure.

File Name: Supplementary Data 15

Description: Bed file of all common CSF-1R broad peaks identified using MACS2 algorithm in monocytes sorted from three healthy donors.

File Name: Supplementary Data 16

Description: Bed file of all common CSF-1R broad peaks identified using MACS2 algorithm in macrophages generated from three healthy donors.

File Name: Supplementary Movie 1

Description: Nuclear localization of CSF-1R holoreceptor is driven by its ligand. Monocytes were differentiated into macrophages with 100ng/mL CSF-1 for 3 days, adherent cells were serumdeprived, their nuclei were stained with Hoechst (blue) and the cells were stimulated 15 min with fluorescent CSF-1 (green) before wash and living cell observation with a spinning microscope (one image every minute).
