# Supplementary material for: Dynamic gene regulation by nuclear colony-stimulating factor 1 receptor in human monocytes and macrophages
Source: Nat Commun. 2019 Apr 26;10:1935. doi: 10.1038/s41467-019-09970-9 (PMC6486619; doi:10.1038/s41467-019-09970-9)
Supplement: Supplementary file 21 — Reporting Summary [file 41467_2019_9970_MOESM21_ESM.pdf]

nature research

Corresponding author(s): Nathalie Droin, PhD
INSERM U1170
Gustave Roussy Cancer Center,
114 rue Edouard Vaillant
94805 Villejuif, France
Phone: 33 1 42 11 63 02
Fax: 33 1 42 11 52 40
email : nathalie.droin@gustaveroussy.fr
or Eric Solary
same address
email : eric.solary@gustaveroussy.fr

Last updated by author(s): Mar 6, 2019

Reporting Summary

Nature Research wishes to improve the reproducibility of the work that we publish. This form provides structure for consistency and transparency in reporting. For further information on Nature Research policies, see Authors & Referees and the Editorial Policy Checklist.

Statistics

For all statistical analyses, confirm that the following items are present in the figure legend, table legend, main text, or Methods section.

n/a	Confirmed	
☐	☒	The exact sample size (n) for each experimental group/condition, given as a discrete number and unit of measurement
☐	☒	A statement on whether measurements were taken from distinct samples or whether the same sample was measured repeatedly
☐	☒	The statistical test(s) used AND whether they are one- or two-sided<br>*Only common tests should be described solely by name; describe more complex techniques in the Methods section.*
☒	☐	A description of all covariates tested
☒	☐	A description of any assumptions or corrections, such as tests of normality and adjustment for multiple comparisons
☐	☒	A full description of the statistical parameters including central tendency (e.g. means) or other basic estimates (e.g. regression coefficient) AND variation (e.g. standard deviation) or associated estimates of uncertainty (e.g. confidence intervals)
☒	☐	For null hypothesis testing, the test statistic (e.g. F, t, r) with confidence intervals, effect sizes, degrees of freedom and P value noted<br>*Give P values as exact values whenever suitable.*
☒	☐	For Bayesian analysis, information on the choice of priors and Markov chain Monte Carlo settings
☒	☐	For hierarchical and complex designs, identification of the appropriate level for tests and full reporting of outcomes
☒	☐	Estimates of effect sizes (e.g. Cohen's d, Pearson's r), indicating how they were calculated

Our web collection on statistics for biologists contains articles on many of the points above.

Software and code

Policy information about availability of computer code

Data collection	SIS MegaviewIII, ImageStream X MK2, imageQuant LAS 4000 camera, Fastq from HiSeq2000 or NovaSeq6000,
Data analysis	Prism, ImageJ, BWA aln v0.7.5a, HOMER v4.9.1, MACS 2.1, IDEAS, deeptools v3.1.2, IGV 2.4.14

For manuscripts utilizing custom algorithms or software that are central to the research but not yet described in published literature, software must be made available to editors/reviewers. We strongly encourage code deposition in a community repository (e.g. GitHub). See the Nature Research guidelines for submitting code & software for further information.

Data

Policy information about availability of data

All manuscripts must include a data availability statement. This statement should provide the following information, where applicable:

- Accession codes, unique identifiers, or web links for publicly available datasets
- A list of figures that have associated raw data
- A description of any restrictions on data availability

E-MTAB-6305 and E-MTAB-7756

Field-specific reporting

Please select the one below that is the best fit for your research. If you are not sure, read the appropriate sections before making your selection.

☒ Life sciences ☐ Behavioural & social sciences ☐ Ecological, evolutionary & environmental sciences

For a reference copy of the document with all sections, see nature.com/documents/nr-reporting-summary-flat.pdf

Life sciences study design

All studies must disclose on these points even when the disclosure is negative.

Sample size	We choosed to use 3 donors for CSF-1R ChIP-seq analyses as 3 biological replicates. These 3 donors are sufficient to identify common reproductive CSF-1R peaks. We used 2 donors for EGR1 ChIPseq as recommended by ENCODE. We used 5 CMML patients for western blot depending on sample availability and the reproductibility of the observation.
Data exclusions	Exclusion of ChIP-seq samples from healthy donors, or CMML patients and one THP-1 cell line with not enough reads after sequencing.
Replication	Fig 1a : 1 fail, Fig 1d : 1 fail with CSF-1R siRNA no fail with blocking peptide, Fig 1e : 1 fail, fig 1f : no fail<br>fig 2a : no fail, fig 2b : no fail, fig 2c : 2 fails, fig 2d : no fail<br>fig 3 : no fail<br>fig 4a : 1 fail, fig 4b : 1 fail in EGR1 ChIP-seq<br>fig 5a : no fail, fig 5b : 1 fail<br>fig 6a : no fail, fig 6b : no fail, fig 6d : no fail<br>fig 7d : 1 fail<br>fig 8 : 1 fail<br>fig 9a : no fail, fig 9b : no fail, fig 9c-d : 2 fails because of no CSF-1R expression
Randomization	N/A
Blinding	Blinding during immunofluorescence quantification

Reporting for specific materials, systems and methods

We require information from authors about some types of materials, experimental systems and methods used in many studies. Here, indicate whether each material, system or method listed is relevant to your study. If you are not sure if a list item applies to your research, read the appropriate section before selecting a response.

Materials & experimental systems

n/a	Involved in the study	
☐	☒	Antibodies
☐	☒	Eukaryotic cell lines
☒	☐	Palaeontology
☒	☐	Animals and other organisms
☐	☒	Human research participants
☒	☐	Clinical data

Methods

n/a	Involved in the study	
☐	☒	ChIP-seq
☒	☐	Flow cytometry
☒	☐	MRI-based neuroimaging

Antibodies

Antibodies used

- CSF-1R antibody, sc-692, santa cruz biotechnology, polyclonal, lot number A0816 K0613 K1212 K0414
- CSF-1R antibody, sc-46662, santa cruz biotechnology, clone B-8, lot number C3012
- CSF-1R antibody, sc-365719, santa cruz biotechnology, clone D-8, lot number D1913 H0515
- CSF-1R antibody, #3152, cell signaling technology, polyclonal, lot number 4
- EGR1 antibody, sc-110, santa cruz biotechnology, clone 588, lot number J0815 I0508
- ELK1 antibody, sc-365876, santa cruz biotechnology, clone E-5, lot number A1617
- YY1 antibody, #2185, cell signaling technology, clone 13G10, lot number 1
- LAMIN B antibody, sc-6217, santa cruz biotechnology, polyclonal, lot number G2116
- B-ACTIN antibody, A5441, sigma aldrich, clone AC-15
- A-TUBULIN, #3873, cell signaling technology, clone DM1A, lot number 8
- H3K4me3, 39159, active motif, polyclonal, lot number 12613005
- H3K4me1, 39297, active motif, polyclonal, lot number 16513009

Validation	- CSF-1R antibody, sc-692, santa cruz biotechnology, polyclonal, lot number A0816 K0613 K1212 K0414, validation by siRNA and blocking peptide for immunofluorescence in human monocytes/macrophages, validation for ChIP-qPCR and ChIP-seq in human monocytes/macrophages, immunofluorescence validation by the supplier (RAW 264.7), PMID : # 24362524, # 20406232, # 19587445, # 16618738, # 12381783, # 10980129, # 9852586<br>- CSF-1R antibody, sc-46662, santa cruz biotechnology, clone B-8, lot number C3012, validation for ChIP-qPCR and ChIP-seq in human monocytes/macrophages, validation by the supplier for western blot and IHC, PMID : # 24362524<br>- CSF-1R antibody, sc-365719, santa cruz biotechnology, clone D-8, lot number D1913 H0515, validation for immunoprecipitation in human monocytes and macrophages, validation by the supplier for western blot and immunofluorescence<br>- CSF-1R antibody, #3152, cell signaling technology, polyclonal, lot number 4, validation for Western blot by siRNA in human monocytes and macrophages, validation by the supplier for western blot, PMID : # 28810630, # 28193701, # 26888110, #26147685, # 25447310, # 24408928, # 23960073, # 21659508, # 20935210<br>- EGR1 antibody, sc-110, santa cruz biotechnology, clone 588, lot number J0815 I0508, validation for ChIP-seq and western blot in human monocytes, validation by the supplier for western blot and immunofluorescence, PMID : # 27384677, # 25378406, # 25201174, # 25147211, # 24391734, # 24060757, # 22428032, # 22552965, # 22035109, # 20453878, # 20514024, # 19681663, # 20204374, # 20018936, # 19847159, # 20130956<br>- ELK1 antibody, sc-365876, santa cruz biotechnology, clone E-5, lot number A1617, validation by the supplier for western blot and IHC<br>- YY1 antibody, #2185, cell signaling technology, clone 13G10, lot number 1, validation for western blot in human monocytes and macrophages, validation by the supplier for western blot, PMID : # 28819251, # 28393842, # 26177460, # 23874387, #22922362, # 20525792<br>- LAMIN B antibody, sc-6217, santa cruz biotechnology, polyclonal, lot number G2116, validation for western blot and immunofluorescence in human monocytes and macrophages, validation by the supplier for western blot, PMID : # 26767983, # 25736587, # 26157010, # 26375988, # 26415859, # 26079946<br>- B-ACTIN antibody, A5441, sigma aldrich, clone AC-15, validation for western blot in human monocytes and macrophages, validation by the supplier for western blot, IF, IHC, PMID : # 15809369, # 15048076, # 21217779, # 20479220, # 22064859<br>- A-TUBULIN, #3873, cell signaling technology, clone DM1A, lot number 8, validation for western blot in human monocytes and macrophages, validation by the supplier for western blot, PMID : # 28924239, # 28744224, # 28534495, # 28427150, # 28356702<br>- H3K4me3, 39159, active motif, polyclonal, lot number 12613005, validation for western blot and ChIP-seq in human monocytes and macrophages, validation by the supplier for western blot, ChIP, ChIP-seq, IF, PMID # 28851877, # 28414795, # 28169375, # 28122508, # 28058014, # 27623009, # 27581382<br>- H3K4me1, 39297, active motif, polyclonal, lot number 16513009, validation for ChIP-seq in human monocytes and macrophages, validation by the supplier for western blot, ChIP, ChIP-seq, IF, PMID # 27992401, # 27106930, # 26752713, # 26229551, # 26230995, # 25666439, # 25747664, # 24732587

Eukaryotic cell lines

Policy information about cell lines

Cell line source(s)	THP-1 and 293T were purchased at ATCC
Authentication	N/A
Mycoplasma contamination	Cell lines were tested for mycoplasma contamination.
Commonly misidentified lines<br>(See ICLAC register)	N/A

Human research participants

Policy information about studies involving human research participants

Population characteristics	28 CMML patients<br>12 males/13 females/3 unknown<br>Mean age 80.3 years (range 60-96)<br>10CMML0/14CMML1/3CMML2/1unknown CMML classification<br>2CMML1 evolved to CMML2, 1 CMML transformed to AML<br>14 untreated, 1 treated with EPO, 3 treated with demethylating agents, 8 treated with hydroxyurea, 2 unknown<br>7 TET2 wt, 20 TET2 mutated<br>17 ASXL1 wt, 10 ASXL1 mutated<br>14 SRSF2 wt, 11 SRSF2 mutated<br>24 NRAS wt, 2 NRAS mutated<br>22 KRAS wt, 2 KRAS mutated<br>22 CBL wt, 2 CBL mutated<br>21 RUNX1 wt, 3 RUNX1 mutated<br>No data available for healthy donors
Recruitment	Newly diagnosed CMML patients whose samples were collected with informed consent.
Ethics oversight	Authorization provided by the ethical committee Ile-de-France 1 (DC-2014-2091)

Note that full information on the approval of the study protocol must also be provided in the manuscript.

ChIP-seq

Data deposition

☒ Confirm that both raw and final processed data have been deposited in a public database such as GEO.

☒ Confirm that you have deposited or provided access to graph files (e.g. BED files) for the called peaks.

Data access links<br>*May remain private before publication.*	ArrayExpress database at EMBL-EBI (www.ebi.ac.uk/arrayexpress) E-MTAB-6306 and E-MTAB-xxxx
Files in database submission	Sample_BC212_CFMS_d0.R1.fastq.gz<br>Sample_BC212_CFMS_d0.bigwig<br>Sample_BC212_CFMS_Md3.R1.fastq.gz<br>Sample_BC212_CFMS_Md3.bigwig<br>Sample_BC212_d0_H3K4me1.R1.fastq.gz<br>Sample_BC212_d0_H3K4me1.bigwig<br>Sample_BC212_H3K4me3_d0.R1.fastq.gz<br>Sample_BC212_H3K4me3_d0.bigwig<br>Sample_BC212_H3K4me3_Md3.R1.fastq.gz<br>Sample_BC212_H3K4me3_Md3.bigwig<br>Sample_BC212_Input_d0.R1.fastq.gz<br>Sample_BC212_Input_d0.bigwig<br>Sample_BC212_Input_Md3.R1.fastq.gz<br>Sample_BC212_Input_Md3.bigwig<br>Sample_BC212_Md3_H3K4me1.R1.fastq.gz<br>Sample_BC212_Md3_H3K4me1.bigwig<br>Sample_BC214_d0_CFMS.R1.fastq.gz<br>Sample_BC214_d0_CFMS.bigwig<br>Sample_BC214_d0_H3K4me3.R1.fastq.gz<br>Sample_BC214_d0_H3K4me3.bigwig<br>Sample_BC214_d0_Input.R1.fastq.gz<br>Sample_BC214_d0_Input.bigwig<br>Sample_BC214_H3K4me1_d0.R1.fastq.gz<br>Sample_BC214_H3K4me1_d0.bigwig<br>Sample_BC214_Md3_CFMS.R1.fastq.gz<br>Sample_BC214_Md3_CFMS.bigwig<br>Sample_BC214_Md3_H3K4me1.R1.fastq.gz<br>Sample_BC214_Md3_H3K4me1.bigwig<br>Sample_BC214_Md3_H3K4me3.R1.fastq.gz<br>Sample_BC214_Md3_H3K4me3.bigwig<br>Sample_BC214_Md3_Input.R1.fastq.gz<br>Sample_BC214_Md3_Input.bigwig<br>Sample_BC215_d0_CFMS.R1.fastq.gz<br>Sample_BC215_d0_CFMS.bigwig<br>Sample_BC215_d0_H3K4me1.R1.fastq.gz<br>Sample_BC215_d0_H3K4me1.bigwig<br>Sample_BC215_d0_H3K4me3.R1.fastq.gz<br>Sample_BC215_d0_H3K4me3.bigwig<br>Sample_BC215_d0_input.R1.fastq.gz<br>Sample_BC215_d0_input.bigwig<br>Sample_BC215_Md3_CFMS.R1.fastq.gz<br>Sample_BC215_Md3_CFMS.bigwig<br>Sample_BC215_Md3_H3K4me1.R1.fastq.gz<br>Sample_BC215_Md3_H3K4me1.bigwig<br>Sample_BC215_Md3_H3K4me3.R1.fastq.gz<br>Sample_BC215_Md3_H3K4me3.bigwig<br>Sample_BC215_Md3_Input.R1.fastq.gz<br>Sample_BC270_14p_j0_CFMS.R1.fastq.gz<br>Sample_BC270_14p_j0_CFMS.bigwig<br>Sample_BC270_14p_j0_input.R1.fastq.gz<br>Sample_BC270_14p_j0_input.bigwig<br>Sample_BC275_J0_EGRI.R1.fastq.gz<br>Sample_BC275_J0_EGRI.bigwig<br>Sample_BC275_J0_input.R1.fastq.gz<br>Sample_BC275_J0_input.bigwig<br>Sample_BC276_J0_EGRI.R1.fastq.gz<br>Sample_BC276_J0_EGRI.bigwig<br>Sample_BC276_J0_input.R1.fastq.gz<br>Sample_BC276_J0_input.bigwig<br>Sample_BC313_14p_j0_CFMS.R1.fastq.gz<br>Sample_BC313_14p_j0_CFMS.bigwig<br>Sample_BC313_14p_j0_H3K4me3.R1.fastq.gz

Sample_BC313_14p_j0_H3K4me3.bigwig
Sample_BC313_14p_j0_input.R1.fastq.gz
Sample_BC313_14p_j0_input.bigwig
Sample_BC313_14p_m6h_CFMS.R1.fastq.gz
Sample_BC313_14p_m6h_CFMS.bigwig
Sample_BC313_14p_m6h_H3K4me3.R1.fastq.gz
Sample_BC313_14p_m6h_H3K4me3.bigwig
Sample_BC313_14p_m6h_input.R1.fastq.gz
Sample_BC313_14p_m6h_input.bigwig
Sample_CMML1941_CFMS_J1.R1.fastq.gz
Sample_CMML1941_CFMS_J1.bigwig
Sample_CMML1941_Input.R1.fastq.gz
Sample_CMML1941_Input.bigwig
Sample_cmml1982_14p_j0_CFMS.R1.fastq.gz
Sample_cmml1982_14p_j0_CFMS.bigwig
Sample_cmml1982_14p_j0_input.R1.fastq.gz
Sample_cmml1982_14p_j0_input.bigwig

THP-1_clone_31_input.R1.fastq.gz
THP-1_clone_31_input.R2.fastq.gz
THP-1_clone_31_input.bigwig
THP-1_clone_31_ChIP_CFMS.R1.fastq.gz
THP-1_clone_31_ChIP_CFMS.R2.fastq.gz
THP-1_clone_31_ChIP_CFMS.bigwig
THP-1_clone_30_input.R1.fastq.gz
THP-1_clone_30_input.R2.fastq.gz
THP-1_clone_30_input.bigwig
THP-1_clone_30_ChIP_CFMS.R1.fastq.gz
THP-1_clone_30_ChIP_CFMS.R2.fastq.gz
THP-1_clone_30_ChIP_CFMS.bigwig
THP-1_clone_21_input.R1.fastq.gz
THP-1_clone_21_input.R2.fastq.gz
THP-1_clone_21_input.bigwig
THP-1_clone_21_ChIP_CFMS.R1.fastq.gz
THP-1_clone_21_ChIP_CFMS.R2.fastq.gz
THP-1_clone_21_ChIP_CFMS.bigwig
THP-1_clone_18_input.R1.fastq.gz
THP-1_clone_18_input.R2.fastq.gz
THP-1_clone_18_input.bigwig
THP-1_clone_18_ChIP_CFMS.R1.fastq.gz
THP-1_clone_18_ChIP_CFMS.R2.fastq.gz
THP-1_clone_18_ChIP_CFMS.bigwig
THP-1_clone_11_input.R1.fastq.gz
THP-1_clone_11_input.R2.fastq.gz
THP-1_clone_11_input.bigwig
THP-1_clone_11_ChIP_CFMS.R1.fastq.gz
THP-1_clone_11_ChIP_CFMS.R2.fastq.gz
THP-1_clone_11_ChIP_CFMS.bigwig
THP-1_clone_11-18-21_input.R1.fastq.gz
THP-1_clone_11-18-21_input.R2.fastq.gz
THP-1_clone_11-18-21_input.bigwig
THP-1_clone_11-18-21_ChIP_CFMS.R1.fastq.gz
THP-1_clone_11-18-21_ChIP_CFMS.R2.fastq.gz
THP-1_clone_11-18-21_ChIP_CFMS.bigwig
CMML2130_CFMS.R1.fastq.gz
CMML2130_CFMS.R2.fastq.gz
CMML2130_CFMS.bigwig
CMML2130_input.R1.fastq.gz
CMML2130_input.R2.fastq.gz
CMML2130_input.bigwig
CMML2609_CFMS.R1.fastq.gz
CMML2609_CFMS.R2.fastq.gz
CMML2609_CFMS.bigwig
CMML2609_input.R1.fastq.gz
CMML2609_input.R2.fastq.gz
CMML2609_input.bigwig

Genome browser session (e.g. UCSC)

no longer applicable

Methodology

Replicates

We choosed to use 3 donors for CSF-1R, H3K4me3, H3K4me1 ChIP-seq analyses as 3 biological replicates. These 3 donors are sufficient to identify common CSF-1R reproductive peaks. We used 2 donors for EGR1 ChIPseq as recommended by ENCODE for transcription factors.

Sequencing depth

Sample name, total number of reads, uniquely mapped reads, length of reads and whether they were paired- or single-end
Sample_BC212_CFMS_d0, 2.12E+07 total reads, 1.16E+07 uniquely mapped, 50, single-end
Sample_BC212_CFMS_Md3, 7.71E+07 total reads, 3.01E+07 uniquely mapped, 50, single-end
Sample_BC212_d0_H3K4me1, 7.86E+07 total reads, 4.47E+07 uniquely mapped, 50, single-end
Sample_BC212_H3K4me3_d0, 4.03E+07 total reads, 1.33E+07 uniquely mapped, 50, single-end
Sample_BC212_H3K4me3_Md3, 4.14E+07 total reads, 2.66E+07 uniquely mapped, 50, single-end
Sample_BC212_Input_d0, 8.22E+07 total reads, 7.07E+07 uniquely mapped, 50, single-end
Sample_BC212_Input_Md3, 1.04E+08 total reads, 9.37E+07 uniquely mapped, 50, single-end
Sample_BC212_Md3_H3K4me1, 1.20E+07 total reads, 8.05E+06 uniquely mapped, 50, single-end
Sample_BC214_d0_CFMS, 3.77E+07 total reads, 2.35E+07 uniquely mapped, 50, single-end
Sample_BC214_d0_H3K4me3, 4.53E+07 total reads, 2.74E+07 uniquely mapped, 50, single-end
Sample_BC214_d0_Input, 8.63E+07 total reads, 7.69E+07 uniquely mapped, 50, single-end
Sample_BC214_H3K4me1_d0, 1.21E+08 total reads, 6.66E+07 uniquely mapped, 50, single-end
Sample_BC214_Md3_CFMS, 2.48E+07 total reads, 1.92E+07 uniquely mapped, 50, single-end
Sample_BC214_Md3_H3K4me1, 8.54E+07 total reads, 6.34E+07 uniquely mapped, 50, single-end
Sample_BC214_Md3_H3K4me3, 2.03E+07 total reads, 1.53E+07 uniquely mapped, 50, single-end
Sample_BC214_Md3_Input, 5.58E+07 total reads, 4.80E+07 uniquely mapped, 50, single-end
Sample_BC215_d0_CFMS, 5.26E+07 total reads, 3.59E+07 uniquely mapped, 50, single-end
Sample_BC215_d0_H3K4me1, 1.05E+08 total reads, 5.70E+07 uniquely mapped, 50, single-end
Sample_BC215_d0_H3K4me3, 3.49E+07 total reads, 2.38E+07 uniquely mapped, 50, single-end
Sample_BC215_d0_input, 6.00E+07 total reads, 5.44E+07 uniquely mapped, 50, single-end
Sample_BC215_Md3_CFMS, 3.69E+07 total reads, 2.72E+07 uniquely mapped, 50, single-end
Sample_BC215_Md3_H3K4me1, 3.40E+07 total reads, 2.42E+07 uniquely mapped, 50, single-end
Sample_BC215_Md3_H3K4me3, 2.79E+07 total reads, 1.95E+07 uniquely mapped, 50, single-end
Sample_BC215_Md3_Input, 9.07E+07 total reads, 8.15E+07 uniquely mapped, 50, single-end
Sample_BC270_14p_j0_CFMS, 6.13E+07 total reads, 3.62E+07 uniquely mapped, 50, single-end
Sample_BC270_14p_j0_input, 2.37E+07 total reads, 2.14E+07 uniquely mapped, 50, single-end
Sample_BC275_J0_EGRI, 2.19E+08 total reads, 4.29E+07 uniquely mapped, 50, single-end
Sample_BC275_J0_input, 4.91E+07 total reads, 2.78E+07 uniquely mapped, 50, single-end
Sample_BC276_J0_EGRI, 5.53E+07 total reads, 1.43E+07 uniquely mapped, 50, single-end
Sample_BC276_J0_input, 5.49E+07 total reads, 3.35E+07 uniquely mapped, 50, single-end
Sample_BC313_14p_j0_CFMS, 1.04E+08 total reads, 7.22E+07 uniquely mapped, 50, single-end
Sample_BC313_14p_j0_H3K4me3, 9.80E+07 total reads, 6.92E+07 uniquely mapped, 50, single-end
Sample_BC313_14p_j0_input, 5.50E+07 total reads, 4.44E+07 uniquely mapped, 50, single-end
Sample_BC313_14p_m6h_CFMS, 3.20E+07 total reads, 2.24E+07 uniquely mapped, 50, single-end
Sample_BC313_14p_m6h_H3K4me3, 3.41E+07 total reads, 2.20E+07 uniquely mapped, 50, single-end
Sample_BC313_14p_m6h_input, 1.59E+07 total reads, 1.46E+07 uniquely mapped, 50, single-end
Sample_CMML1941_CFMS_J1, 2.38E+08 total reads, 1.29E+08 uniquely mapped, 50, single-end
Sample_CMML1941_Input.R1, 8.01E+07 total reads, 6.82E+07 uniquely mapped, 50, single-end
Sample_cmml1982_14p_j0_CFMS, 5.95E+07 total reads, 3.64E+07 uniquely mapped, 50, single-end
Sample_cmml1982_14p_j0_input, 1.52E+07 total reads, 1.25E+07 uniquely mapped, 50, single-end

THP-1_clone_31_input, 5.86E+07 total reads, 2.74E+07 uniquely mapped, 50, paired-end
THP-1_clone_31_ChIP_CFMS, 2.77E+07 total reads, 1.27E+07 uniquely mapped, 50, paired-end
THP-1_clone_30_input, 5.27E+07 total reads, 2.82E+07 uniquely mapped, 50, paired-end
THP-1_clone_30_ChIP_CFMS, 5.38E+07 total reads, 2.40E+07 uniquely mapped, 50, paired-end
THP-1_clone_21_input, 6.19E+07 total reads, 2.79E+07 uniquely mapped, 50, paired-end
THP-1_clone_21_ChIP_CFMS, 4.02E+06 total reads, 1.79E+06 uniquely mapped, 50, paired-end
THP-1_clone_18_input, 2.88E+07 total reads, 1.32E+07 uniquely mapped, 50, paired-end
THP-1_clone_18_ChIP_CFMS, 7.45E+06 total reads, 3.20E+06 uniquely mapped, 50, paired-end
THP-1_clone_11_input, 2.11E+07 total reads, 9.72E+06 uniquely mapped, 50, paired-end
THP-1_clone_11_ChIP_CFMS, 7.84E+06 total reads, 3.60E+06 uniquely mapped, 50, paired-end
THP-1_clone_11-18-21_ChIP_CFMS, 1.93E+07 total reads, 8.59E+06 uniquely mapped, 50, paired-end
CMML2130_CFMS, 9.20E+07 total reads, 5.25E+07 uniquely mapped, 50, paired-end

CMML2130_input, 1.29E+08 total reads, 7.55E+07 uniquely mapped, 50, paired-end
CMML2609_CFMS, 7.51E+07 total reads, 4.37E+07 uniquely mapped, 50, paired-end
CMML2609_input, 2.11E+08 total reads, 1.17E+08 uniquely mapped, 50, paired-end

Antibodies

CSF-1R antibody, sc-46662, santa cruz biotechnology, clone B-8, lot number C3012
H3K4me3, 39159, active motif, polyclonal, lot number 12613005
H3K4me1, 39297, active motif, polyclonal, lot number 16513009
CSF-1R antibody, sc-692, santa cruz biotechnology, polyclonal, lot number A0816 K0613 K1212 K0414

Peak calling parameters

To analyze our ChIP-seq data, we used MACS2 algorithm, selecting the broad option given the profile of CSF1R binding (more details are given below), not building the shifting model (using extsize, whose choice was based on our library size) and using the default q-value cut off (all the detailed parameters used for the analysis are provided in the revised Material and Method).
Reads were aligned into human genome hg19 with BWA aln (v0.7.5a). Peak calling for H3K4me3 and EGR1 was performed using MACS 2.1 using default options for narrow peaks. For CSF-1R and H3K4me1 ChIP-seq analysis, MACS2.1 was used using the following parameters: --nomodel --broad --extsize 200. Annotation and motif analyses have been done with HOMER (v4.9.1) using a window of 600bp around peak summit. Bigwig files normalized for sequencing depths have been generated with deeptools v3.1.2. Integrative Genomics Viewer (IGV 2.4.14) was used for representation. Heatmaps of short read coverage across the genomic regions of interest (TSS, peaks) were done using deeptools v3.1.2.

Data quality

Following the reviewer’s advice, we performed IDR analysis on these peaks using ENCODE3 guidelines (using replicates, pooled replicate, pseudo-replicates and pooled pseudo-replicates), which identified an optimal set of 3,054 peaks in monocytes. The overlap between common peaks in the 3 replicates and IDR optimal set was 2,303 peaks, including all the peaks subsequently characterized in our studies.
Script used for IDR

```
#!/bin/bash

REP1=[name of replicate #1]
REP2=[name of replicate #2]
REP3=[name of replicate #3]
REP1_IP_BAM_FILE=[file name of replicate #1 IP BAM alignement]
REP2_IP_BAM_FILE=[file name of replicate #2 IP BAM alignement]
REP3_IP_BAM_FILE=[file name of replicate #3 IP BAM alignement]
REP1_Input_BAM_FILE=[file name of replicate #1 input BAM alignement]
REP2_Input_BAM_FILE=[file name of replicate #2 input BAM alignement]
REP3_Input_BAM_FILE=[file name of replicate #3 input BAM alignement]

# =============================
# Split BAM files to generate
# pseudo replicates.
# =============================
split_bam.py -b ${REP1_IP_BAM_FILE} -o1 "${REP1}"_PR1.bam -o2 "${REP1}"_PR2.bam -p 0.5
split_bam.py -b ${REP2_IP_BAM_FILE} -o1 "${REP2}"_PR1.bam -o2 "${REP2}"_PR2.bam -p 0.5
split_bam.py -b ${REP3_IP_BAM_FILE} -o1 "${REP3}"_PR1.bam -o2 "${REP3}"_PR2.bam -p 0.5

# =============================
# Perform MACS2 peakcalling.
# =============================
# =============================
# On true replicates.
# =============================
macs2 callpeak -t ${REP1_IP_BAM_FILE} -c ${REP1_Input_BAM_FILE} -g hs -n MACS2_"${REP1}"_BROAD -f BAM --nomodel --
broad &
macs2 callpeak -t ${REP2_IP_BAM_FILE} -c ${REP2_Input_BAM_FILE} -g hs -n MACS2_"${REP2}"_BROAD -f BAM --nomodel --
broad &
macs2 callpeak -t ${REP3_IP_BAM_FILE} -c ${REP3_Input_BAM_FILE} -g hs -n MACS2_"${REP3}"_BROAD -f BAM --nomodel --
broad &&

wait

# =============================
# On pooled true replicates.
# =============================
macs2 callpeak -t ${REP1_IP_BAM_FILE} ${REP2_IP_BAM_FILE} ${REP3_IP_BAM_FILE} -c ${REP1_Input_BAM_FILE}
${REP2_Input_BAM_FILE} ${REP3_Input_BAM_FILE} -g hs -n MACS2_POOLED_BROAD -f BAM --nomodel --broad

# =============================
# On pseudo replicates.
# =============================
macs2 callpeak -t "${REP1}"_PR1.bam -c ${REP1_Input_BAM_FILE} -g hs -n MACS2_"${REP1}"_BROAD_pr1 -f BAM --nomodel
--broad &
macs2 callpeak -t "${REP1}"_PR2.bam -c ${REP1_Input_BAM_FILE} -g hs -n MACS2_"${REP1}"_BROAD_pr2 -f BAM --nomodel
--broad &
```

```
macs2 callpeak -t "${REP2}"_PR1.bam -c ${REP2_Input_BAM_FILE} -g hs -n MACS2_"${REP2}"_BROAD_pr1 -f BAM --nomodel
--broad &
macs2 callpeak -t "${REP2}"_PR2.bam -c ${REP2_Input_BAM_FILE} -g hs -n MACS2_"${REP2}"_BROAD_pr2 -f BAM --nomodel
--broad &

macs2 callpeak -t "${REP3}"_PR1.bam -c ${REP3_Input_BAM_FILE} -g hs -n MACS2_"${REP3}"_BROAD_pr1 -f BAM --nomodel
--broad &
macs2 callpeak -t "${REP3}"_PR2.bam -c ${REP3_Input_BAM_FILE} -g hs -n MACS2_"${REP3}"_BROAD_pr2 -f BAM --nomodel
--broad &&

wait

# ============================
# On pooled pseudo replicates.
# ============================
macs2 callpeak -t "${REP1}"_PR1.bam "${REP2}"_PR1.bam "${REP3}"_PR1.bam -c ${REP1_Input_BAM_FILE}
${REP2_Input_BAM_FILE} ${REP3_Input_BAM_FILE} -g hs -n MACS2_BROAD_PPR1 -f BAM --nomodel --broad &
macs2 callpeak -t "${REP1}"_PR2.bam "${REP2}"_PR2.bam "${REP3}"_PR2.bam -c ${REP1_Input_BAM_FILE}
${REP2_Input_BAM_FILE} ${REP3_Input_BAM_FILE} -g hs -n MACS2_BROAD_PPR2 -f BAM --nomodel --broad &&

wait

gzip -k *broadPeak

#source activate idr_env

REP1_PEAK_FILE="MACS2_"${REP1}"_BROAD_peaks.broadPeak"
REP2_PEAK_FILE="MACS2_"${REP2}"_BROAD_peaks.broadPeak"
REP3_PEAK_FILE="MACS2_"${REP3}"_BROAD_peaks.broadPeak"
REP1_PR1_PEAK_FILE="MACS2_"${REP1}"_BROAD_pr1_peaks.broadPeak"
REP1_PR2_PEAK_FILE="MACS2_"${REP1}"_BROAD_pr2_peaks.broadPeak"
REP2_PR1_PEAK_FILE="MACS2_"${REP2}"_BROAD_pr1_peaks.broadPeak"
REP2_PR2_PEAK_FILE="MACS2_"${REP2}"_BROAD_pr2_peaks.broadPeak"
REP3_PR1_PEAK_FILE="MACS2_"${REP3}"_BROAD_pr1_peaks.broadPeak"
REP3_PR2_PEAK_FILE="MACS2_"${REP3}"_BROAD_pr2_peaks.broadPeak"
PPR1_PEAK_FILE="MACS2_BROAD_PPR1_peaks.broadPeak"
PPR2_PEAK_FILE="MACS2_BROAD_PPR2_peaks.broadPeak"
POOLED_PEAK_FILE="MACS2_POOLED_BROAD_peaks.broadPeak"
BLACKLIST="wgEncodeDacMapabilityConsensusExcludable.bed.gz"
IDR_THRESH=0.05

# ============================
# Perform IDR analysis.
# Generate a plot and IDR output with additional columns including IDR scores.
# ============================
idr --samples ${REP1_PEAK_FILE} ${REP2_PEAK_FILE} --peak-list ${POOLED_PEAK_FILE} --input-file-type broadPeak --output-
file IDR_"${REP1}"_VS_"${REP2}" --rank signal.value --soft-idr-threshold ${IDR_THRESH} --plot --use-best-multisummit-IDR
2>IDR.out
idr --samples ${REP1_PEAK_FILE} ${REP3_PEAK_FILE} --peak-list ${POOLED_PEAK_FILE} --input-file-type broadPeak --output-
file IDR_"${REP1}"_VS_"${REP3}" --rank signal.value --soft-idr-threshold ${IDR_THRESH} --plot --use-best-multisummit-IDR
2>>IDR.out
idr --samples ${REP2_PEAK_FILE} ${REP3_PEAK_FILE} --peak-list ${POOLED_PEAK_FILE} --input-file-type broadPeak --output-
file IDR_"${REP2}"_VS_"${REP3}" --rank signal.value --soft-idr-threshold ${IDR_THRESH} --plot --use-best-multisummit-IDR
2>>IDR.out

idr --samples ${REP1_PR1_PEAK_FILE} ${REP1_PR2_PEAK_FILE} --peak-list ${REP1_PEAK_FILE} --input-file-type broadPeak --
output-file IDR_"${REP1}"_PR --rank signal.value --soft-idr-threshold ${IDR_THRESH} --plot --use-best-multisummit-IDR
2>>IDR.out
idr --samples ${REP2_PR1_PEAK_FILE} ${REP2_PR2_PEAK_FILE} --peak-list ${REP2_PEAK_FILE} --input-file-type broadPeak --
output-file IDR_"${REP2}"_PR --rank signal.value --soft-idr-threshold ${IDR_THRESH} --plot --use-best-multisummit-IDR
2>>IDR.out
idr --samples ${REP3_PR1_PEAK_FILE} ${REP3_PR2_PEAK_FILE} --peak-list ${REP3_PEAK_FILE} --input-file-type broadPeak --
output-file IDR_"${REP3}"_PR --rank signal.value --soft-idr-threshold ${IDR_THRESH} --plot --use-best-multisummit-IDR
2>>IDR.out

idr --samples ${PPR1_PEAK_FILE} ${PPR2_PEAK_FILE} --peak-list ${POOLED_PEAK_FILE} --input-file-type broadPeak --output-
file IDR_PPR --rank signal.value --soft-idr-threshold ${IDR_THRESH} --plot --use-best-multisummit-IDR 2>>IDR.out

# ============================
# Get peaks passing IDR threshold of 5%
# ============================
IDR_THRESH_TRANSFORMED=$(awk -v p=${IDR_THRESH} 'BEGIN{print -log(p)/log(10)}')

awk 'BEGIN{OFS="\t"} $11>='"${IDR_THRESH_TRANSFORMED}"' {print $1,$2,$3,$4,$5,$6,$7,$8,$9,$10}'
```

```
IDR_"${REP1}"_VS_"${REP2}" | sort | uniq | sort -k7n,7n | gzip -nc > "${REP1}"_VS_"${REP2}".IDR0.05.broadPeak.gz
NPEAKS_IDR1=$(zcat "${REP1}"_VS_"${REP2}".IDR0.05.broadPeak.gz | wc -l)

awk 'BEGIN{OFS="\t"} $11>='"${IDR_THRESH_TRANSFORMED}"' {print $1,$2,$3,$4,$5,$6,$7,$8,$9,$10}'
IDR_"${REP1}"_VS_"${REP3}" | sort | uniq | sort -k7n,7n | gzip -nc > "${REP1}"_VS_"${REP3}".IDR0.05.broadPeak.gz
NPEAKS_IDR2=$(zcat "${REP1}"_VS_"${REP3}".IDR0.05.broadPeak.gz | wc -l)

awk 'BEGIN{OFS="\t"} $11>='"${IDR_THRESH_TRANSFORMED}"' {print $1,$2,$3,$4,$5,$6,$7,$8,$9,$10}'
IDR_"${REP2}"_VS_"${REP3}" | sort | uniq | sort -k7n,7n | gzip -nc > "${REP2}"_VS_"${REP3}".IDR0.05.broadPeak.gz
NPEAKS_IDR3=$(zcat "${REP2}"_VS_"${REP3}".IDR0.05.broadPeak.gz | wc -l)

awk 'BEGIN{OFS="\t"} $11>='"${IDR_THRESH_TRANSFORMED}"' {print $1,$2,$3,$4,$5,$6,$7,$8,$9,$10}' IDR_"${REP1}"_PR |
sort | uniq | sort -k7n,7n | gzip -nc > "${REP1}"_PR.IDR0.05.broadPeak.gz
NPEAKS_IDR4=$(zcat "${REP1}"_PR.IDR0.05.broadPeak.gz | wc -l)

awk 'BEGIN{OFS="\t"} $11>='"${IDR_THRESH_TRANSFORMED}"' {print $1,$2,$3,$4,$5,$6,$7,$8,$9,$10}' IDR_"${REP2}"_PR |
sort | uniq | sort -k7n,7n | gzip -nc > "${REP2}"_PR.IDR0.05.broadPeak.gz
NPEAKS_IDR5=$(zcat "${REP2}"_PR.IDR0.05.broadPeak.gz | wc -l)

awk 'BEGIN{OFS="\t"} $11>='"${IDR_THRESH_TRANSFORMED}"' {print $1,$2,$3,$4,$5,$6,$7,$8,$9,$10}' IDR_"${REP3}"_PR |
sort | uniq | sort -k7n,7n | gzip -nc > "${REP3}"_PR.IDR0.05.broadPeak.gz
NPEAKS_IDR6=$(zcat "${REP3}"_PR.IDR0.05.broadPeak.gz | wc -l)

awk 'BEGIN{OFS="\t"} $11>='"${IDR_THRESH_TRANSFORMED}"' {print $1,$2,$3,$4,$5,$6,$7,$8,$9,$10}' IDR_PPR | sort |
uniq | sort -k7n,7n | gzip -nc > PPR.IDR0.05.broadPeak.gz
NPEAKS_IDR7=$(zcat PPR.IDR0.05.broadPeak.gz | wc -l)

# ==============================
# Filter using black list
# ==============================
bedtools intersect -v -a "${REP1}"_VS_"${REP2}".IDR0.05.broadPeak.gz -b ${BLACKLIST} | grep -P 'chr[\dXY]+[ \t]' | awk
'BEGIN{OFS="\t"} {if ($5>1000) $5=1000; print $0}' | gzip -nc > "${REP1}"_VS_"${REP2}".IDR0.05.filt.broadPeak.gz
NPEAKS_IDR1_filt=$(zcat "${REP1}"_VS_"${REP2}".IDR0.05.filt.broadPeak.gz | wc -l)

bedtools intersect -v -a "${REP1}"_VS_"${REP3}".IDR0.05.broadPeak.gz -b ${BLACKLIST} | grep -P 'chr[\dXY]+[ \t]' | awk
'BEGIN{OFS="\t"} {if ($5>1000) $5=1000; print $0}' | gzip -nc > "${REP1}"_VS_"${REP3}".IDR0.05.filt.broadPeak.gz
NPEAKS_IDR2_filt=$(zcat "${REP1}"_VS_"${REP3}".IDR0.05.filt.broadPeak.gz | wc -l)

bedtools intersect -v -a "${REP2}"_VS_"${REP3}".IDR0.05.broadPeak.gz -b ${BLACKLIST} | grep -P 'chr[\dXY]+[ \t]' | awk
'BEGIN{OFS="\t"} {if ($5>1000) $5=1000; print $0}' | gzip -nc > "${REP2}"_VS_"${REP3}".IDR0.05.filt.broadPeak.gz
NPEAKS_IDR3_filt=$(zcat "${REP2}"_VS_"${REP3}".IDR0.05.filt.broadPeak.gz | wc -l)

bedtools intersect -v -a "${REP1}"_PR.IDR0.05.broadPeak.gz -b ${BLACKLIST} | grep -P 'chr[\dXY]+[ \t]' | awk
'BEGIN{OFS="\t"} {if ($5>1000) $5=1000; print $0}' | gzip -nc > "${REP1}"_PR.IDR0.05.filt.broadPeak.gz
NPEAKS_IDR4_filt=$(zcat "${REP1}"_PR.IDR0.05.filt.broadPeak.gz | wc -l)

bedtools intersect -v -a "${REP2}"_PR.IDR0.05.broadPeak.gz -b ${BLACKLIST} | grep -P 'chr[\dXY]+[ \t]' | awk
'BEGIN{OFS="\t"} {if ($5>1000) $5=1000; print $0}' | gzip -nc > "${REP2}"_PR.IDR0.05.filt.broadPeak.gz
NPEAKS_IDR5_filt=$(zcat "${REP2}"_PR.IDR0.05.filt.broadPeak.gz | wc -l)

bedtools intersect -v -a "${REP3}"_PR.IDR0.05.broadPeak.gz -b ${BLACKLIST} | grep -P 'chr[\dXY]+[ \t]' | awk
'BEGIN{OFS="\t"} {if ($5>1000) $5=1000; print $0}' | gzip -nc > "${REP3}"_PR.IDR0.05.filt.broadPeak.gz
NPEAKS_IDR6_filt=$(zcat "${REP3}"_PR.IDR0.05.filt.broadPeak.gz | wc -l)

bedtools intersect -v -a PPR.IDR0.05.broadPeak.gz -b ${BLACKLIST} | grep -P 'chr[\dXY]+[ \t]' | awk 'BEGIN{OFS="\t"} {if
($5>1000) $5=1000; print $0}' | gzip -nc > PPR.IDR0.05.filt.broadPeak.gz
NPEAKS_IDR7_filt=$(zcat PPR.IDR0.05.filt.broadPeak.gz | wc -l)
```

Software

Fastq from Hiseq2000 or NovaSeq6000
Integrative Genomics Viewer (IGV 2.4.14)
